# Nominated Texture Based Cervical Cancer Classification

**DOI:** 10.1155/2015/586928

**Published:** 2015-01-14

**Authors:** Edwin Jayasingh Mariarputham, Allwin Stephen

**Affiliations:** ^1^Department of Computer Science & Engineering, Rajaas Engineering College, Vadakkankulam 627116, India; ^2^Department of Computer Science & Engineering, Infant Jesus College of Engineering, Thoothukudi 628851, India

## Abstract

Accurate classification of Pap smear images becomes the challenging task in medical image processing. This can be improved in two ways. One way is by selecting suitable well defined specific features and the other is by selecting the best classifier. This paper presents a nominated texture based cervical cancer (NTCC) classification system which classifies the Pap smear images into any one of the seven classes. This can be achieved by extracting well defined texture features and selecting best classifier. Seven sets of texture features (24 features) are extracted which include relative size of nucleus and cytoplasm, dynamic range and first four moments of intensities of nucleus and cytoplasm, relative displacement of nucleus within the cytoplasm, gray level cooccurrence matrix, local binary pattern histogram, tamura features, and edge orientation histogram. Few types of support vector machine (SVM) and neural network (NN) classifiers are used for the classification. The performance of the NTCC algorithm is tested and compared to other algorithms on public image database of Herlev University Hospital, Denmark, with 917 Pap smear images. The output of SVM is found to be best for the most of the classes and better results for the remaining classes.

## 1. Introduction

Cervical cancer is one of the most common cancers affecting women worldwide and the most common in developing countries [1]. It can be cured, if it is detected in early stages and identify in which stage it belongs and further proper treatment has given in time. At the same time the occurrence and the death rate remains even high in the developing and under developed regions of the world. It is reported that annually 132,000 new cases were diagnosed and 74,000 deaths in India, which is nearly one-third of the global cancer deaths [2]. Screening of cervical cancer can be done by Pap test, which is believed to be the gold standard forever. Due to the subjective disparity of different cytologists, the screening results show more of inconsistencies [3]. The test output shows more of false positive and false negative results, which make the reliability of the screening process a question mark [4]. Also in manual cervical screening process, hundreds of images are analyzed daily; the classification of cells become tough and the possibility of human errors become high.

Many automatic and semiautomatic methods have been proposed in various times to detect various stages of cervical cancer. Many of these methods were not supported in achieving the objectives of providing measured variables which could eliminate the interpretation errors and interobserver discrepancy [5]. Pap smear images are rich in various features like color, shape, and texture. The process of accurate extraction of unique visual features from these images would very well help in developing an automated screening device. This can be achieved only through texture feature than others since all the cellular changes are observed only using these features. Since the texture parameters are the simple mathematical representations like smooth, rough, and grainy, then the analysis becomes easier [6]. Analyzing all the above issues, two important challenges are considered. First, selection of unique texture features suitable for classification. Second, selection of the most efficient and scalable classifier improves the accuracy more.

Plissiti et al. [7] have developed the fully automated method to detect the nucleus in Pap smear images. Using morphological analysis, the nuclei centroids are detected and by applying distance dependent rule and classification algorithms on resulted centroids, the undesirable artifacts were removed from the cell.

By considering nucleus as the most informative region of the cell, Sobrevilla et al. [8] have been presented an algorithm for automatic nuclei detection of cytology cell. This algorithm combines color, cytopathologists knowledge, and fuzzy systems which show high performance and more computational speed. Harandi et al. [9] have developed a system for the detection of cytoplasm and nucleus from ThinPrep images. The geometric active contours were used as the segmentation tool. In this method, localization of cell objects were done in low resolution and boundary detection of cytoplasm and nucleus were done in high resolution. Bergmeir et al. [10] have developed an algorithm used to detect cell nuclei and cytoplasm. This algorithm used the combination of voting scheme and prior knowledge to locate the cell nuclei and elastic segmentation to determine the shape of nucleus. The noise is removed with mean-shift and median filters, and edges were extracted with canny edge detection algorithm.

Most of the segmentation methods discussed in this literature focused on nucleus and cytoplasm extraction, which requires higher contrast around the boundaries of nucleus. The heavily stained cervical smears, overlapping of cell images and blurred images to overexposing or underexposing of light in microscope even cause difficulties in segmentation [11].

The automatic classification of Pap smear images focuses on marking of single cells into any one of binary classes (normal and abnormal) or multiple classes (based on severity). Multiple classification of Pap smear images became popular when CIN based classification on cervical cytology images was proposed. Holmquist et al. [12] developed a binary classification method to distinguish between normal and abnormal cells. The dual wavelength method was used for the automatic isolation of nucleus from cytoplasm. The classification procedures were done based on the extraction of density-oriented, shape-oriented, and texture-oriented parameters. Chou and Shapiro [13] have proposed a method used hierarchical multiple classifier scheme. This method used graph-theoretic clustering algorithm to group the training data, component classifiers as the inputs to a super-classifier, and subclass labeling is used to improve the classification accuracy. Marinakis et al. [14] proposed a metaheuristic approach to classify the Pap smear cells. Uniquely described twenty features are extracted from each cell image and classified as normal and abnormal type. The genetic algorithm is used to find the best possible performing subset selection.

Most of the medical image classification methods supervised learning algorithms which challenges to find out the connection between the independent and dependent variables. Support vector machine (SVM) and neural network (NN) are the most promising methods of this category. Support vector machine (SVM) is a supervised classification method introduced by Cortes and Vapnik [15] in 1992. SVM plays vital role in cytology image analysis including yeast cells on suspension in bioreactors [16], cells in culture using microscopy [17], and cells on sections of brain tumors [18]. NN classifiers are based on statistical probabilities in making classification decisions [19]. NN uses the training set which contains inputs, outputs, and the learning rules.

Chen et al. [20] have developed an algorithm for segmenting nucleus and cytoplasm counters. This system classifies the Pap smear cells into anyone of four different types of classes using SVM. Two experiments were conducted to validate the classification performance which showed the best performance outputs. Mat-Isa et al. [21] have proposed an automatic cervical cancer diagnostic system based on hierarchical hybrid multilayered perceptron network. In this method, region-growing-based algorithm is used for the feature extraction procedure and classification is done by NN.

The above literatures clearly show that none of the single texture feature is suitable in improving classification accuracy. The necessity of scalable and cost effective algorithm is required to improve the efficiency of the classification system. Our classification method NTCC classifies the Pap smear cells into anyone of seven stages, based on the work done by researchers on the public database of Herlev University Hospital, Denmark [22, 23]. The detailed description of Pap smear cells is shown in Table 1. By carefully extracting 24 features, the images are classified into any one of seven classes, namely, superficial squamous, intermediate squamous, columnar, mild dysplasia, moderate dysplasia, severe dysplasia, and carcinoma in situ. Already many researchers have contributed various classification algorithms based on this public database [14, 24].

## 2. Nominated Texture Based Cervical Cancer (NTCC) Classification System

**Figure 1:**
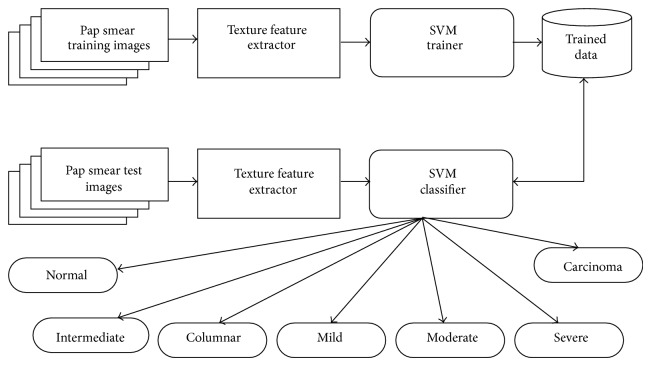
Architecture of the proposed system.

The NTCC classification system consists of texture feature extractor, SVM trainer, SVM classifier, and the database to store the trained and stored database. The architecture of the proposed classification system is depicted in Figure 1. The system involves the following steps: preparation of Pap smear images and preprocessing, segmentation of nucleus and cytoplasm, extraction of texture features, and classification.

### 2.1. Preparation and Preprocessing of Pap Smear Images

The cytology images are acquired through a powerful microscope by the skilled cytotechnicians. All images were captured with a resolution of 0.201 *μ*m/pixel from the public database of cervical cancer, Herlev University Hospital, Denmark [23]. The purpose of the preprocessing step is to suppress the unwanted noise found in cervical image samples and enhances them for further processing. In general the nucleus region of the cervical cytology cell has larger distribution of darker pixel than cytoplasm. In this step the input images are first inverted, and then the image binarization followed by morphological closing operation with structuring element of five has been performed. The rough segmentation of cell nucleus can be done through morphological filling operation.

### 2.2. Feature Extraction and Selection

Feature selection chooses the optimum subset of features from the enormous set of potentially useful features which may be available in a given problem domain. By selecting the precise number of features, it is able to reduce the storage space and computational time, which will certainly improve the performance [25, 26]. Also too many dimensions in the feature space may drastically increase the computational complexity and deteriorate the discriminating power of the feature set due to the distortion and noise [27].

In cervical cancer classification system seven sets of features are extracted. They are relative size of nucleus and cytoplasm, dynamic range and first four moments of intensities of nucleus and cytoplasm, relative displacement of nucleus within the cytoplasm, gray level cooccurrence matrix features, local binary pattern histogram, tamura features, and edge orientation histogram which includes the total features of 24.

(1) The size of nucleus and cytoplasm plays a vital role in classifying the cervical cell type:

(1) $$\begin{array}{c} \frac{\text{N}}{\text{C}}=\frac{{\text{Nucleus}}_{\text{area}}}{{\text{Nucleus}}_{\text{area}}+{\text{Cytoplasm}}_{\text{area}}}, \end{array}$$

where Nucleus_area_ is the proportion of number of pixels of nucleus (N) and Cytoplasm_area_ is the proportion of number of pixels of cytoplasm (C).

(2) Dynamic range of an image and the first four moments of the intensities of nucleus and cytoplasm provides four dissimilar statistical moments. They are calculated by individual values of image pixels and not on the cooccurrence of neighboring pixel values. Dynamic range (dr) of an image is the difference between the intensity values of the brightest and the darkest pixel in the image. The four moments are mean (*m*), variance (*σ* ^2^), skewness (*μ*_3_(*z*)), and Kurtosis (*μ*_4_) as follows [28]:

(2) $$\begin{array}{c} \text{dr}={\text{Intensity}}_{\max}-{\text{Intensity}}_{\min},\\ m=\overset{L_{}-1}{\underset{k_{}=0}{\sum }}z_{k}p\left(z_{k}\right),\\ \sigma^{2}=\overset{L_{\;}-1}{\underset{k_{\;}=0}{\sum }}{\left(z_{k}-m\right)}^{2}p\left(z_{k}\right),\\ \mu_{3}\left(z\right)=\overset{L-1}{\underset{i=0}{\sum }}{\left(z_{k}-m\right)}^{3}p\left(z_{k}\right),\\ \mu_{4}=\sigma^{-4}\overset{N_{L}-1}{\underset{z_{i}=0}{\sum }}{\left(z_{i}-\mu \right)}^{4}p\left(z_{i}\right)-3. \end{array}$$

(3) The movements position of nucleus inside the cytoplasm help in classifying stages. This can be achieved by extracting the relative displacement of nucleus within the cytoplasm:

(3) $$\begin{array}{c} {\text{Dist}}_{\text{cyto-nucl}}={\text{Cyto}}_{\text{centroid}}-{\text{Nucl}}_{\text{centroid}}, \end{array}$$

where Cyto_centroid_ is the centroid of cytoplasm and Nucl_centroid_ is the centroid of nucleus.

Relative displacement can be calculated by

(4) $$\begin{array}{c} \text{RD}=\frac{{\text{Dist}}_{\text{cyto-nucl}}}{\text{Cytoplasm}\;\text{Diameter}}. \end{array}$$

(4) Haralick's gray-level cooccurrence matrices (GLCMs) have been used very successfully in texture classification [29]. Out of 14 features outlined, we considered first 11 texture features suitable for our experiment.

(a) Angular second moment:
  (5) $$\begin{array}{c} Hf_{1}=\overset{j}{\underset{i}{\sum }}{\left\{p\left(i,j\right)\right\}}^{2}. \end{array}$$
(b) Contrast:
  (6) $$\begin{array}{c} {Hf}_{2}=\overset{N_{g}-1}{\underset{n=0}{\sum }}n^{2}\left\{\overset{N_{g}}{\underset{i=1}{\sum }}\;\overset{N_{g}}{\underset{j=1}{\sum }}p\left(i,j\right)\right\};\;\left|i-j\right|=n. \end{array}$$
(c) Correlation:
  (7) $$\begin{array}{c} {Hf}_{3}=\frac{\sum_{i}^{}\sum_{j}^{}\left(ij\right)p\left(i,j\right)-\mu_{x}\mu_{y}}{\sigma_{x}\sigma_{y}}. \end{array}$$
(d) Sum of squares:
  (8) $$\begin{array}{c} {Hf}_{4}=\underset{i}{\sum }\underset{j}{\sum }{\left(i-\mu \right)}^{2}p\left(i,j\right). \end{array}$$
(e) Inverse difference moment:
  (9) $$\begin{array}{c} {Hf}_{5}=\overset{}{\underset{i}{\sum }}\overset{}{\underset{j}{\sum }}\frac{i}{1+{\left(i-j\right)}^{2}}p\left(i,j\right). \end{array}$$
(f) Sum average:
  (10) $$\begin{array}{c} {Hf}_{6}=\overset{{2N}_{g}}{\underset{i_{}=2}{\sum }}i_{}p_{x+y}\left(i\right). \end{array}$$
(g) Sum variance:
  (11) $$\begin{array}{c} {Hf}_{7}=\overset{{2N}_{g}}{\underset{i=2}{\sum }}{\left(i-f_{8}\right)}^{2}p_{x+y}\left(i\right). \end{array}$$
(h) Sum entropy:
  (12) $$\begin{array}{c} {Hf}_{8}=-\overset{{2N}_{g}}{\underset{i_{}=2}{\sum }}p_{x+y}\left(i\right)\log\left\{p_{x+y}\left(i\right)\right\}. \end{array}$$
(i) Entropy:
  (13) $$\begin{array}{c} Hf_{9}=-\overset{N_{g}-1}{\underset{i=0}{\sum }}\;\overset{N_{g}-1}{\underset{j=0}{\sum }}p_{i,j}{\log p}_{i,j}. \end{array}$$
(j) Difference variance:
  (14) $$\begin{array}{c} Hf_{10}=\text{variance}\;\text{of}\;p_{x-y}. \end{array}$$
(k) Difference entropy:
  (15) $$\begin{array}{c} {Hf}_{11}=\overset{N_{g}-1}{\underset{i=0}{\sum }}p_{x-y}\left(i\right)\log\left\{p_{x-y}\left(i\right)\right\}. \end{array}$$

(5) Local binary pattern (LBP) [30] transforms an image into an array or integer labels. It is computed by comparing a given pixel with its neighbors:

(16) $$\begin{array}{c} {\text{LBP}}_{P,R}\left(x,y\right)=\overset{P-1}{\underset{p=0}{\sum }}s\left(g_{p}-g_{c}\right)2^{P},\;\;s\left(x\right)=\left\{\begin{array}{cc} 1, & x\geq 0,\\ 0, & x<0, \end{array}\right. \end{array}$$

where *g*_*p*_ − *g*_*c*_ is variant to changes of the mean gray value of the image.

(6) Tamura's texture features like coarseness, contrast, and directionality [31] are extracted which are purely based on human visual perception are extracted for our experiment.

(a) Coarseness:
  (17) $$\begin{array}{c} F_{\text{crs}}=\frac{1}{n\times m}\overset{n}{\underset{i}{\sum }}\overset{m}{\underset{j}{\sum }}S_{\max}\left(i,j\right). \end{array}$$
(b) Contrast:
  (18) $$\begin{array}{c} F_{\text{con}}=\frac{\sigma }{{\left(\propto_{4}\right)}^{n}},\;\text{where}\;\;\propto_{4}=\frac{\mu_{4}}{\sigma^{4}}. \end{array}$$
(c) Directionality:
  (19) $$\begin{array}{c} F_{\text{dir}}=\overset{n_{p}}{\underset{p}{\sum }}\overset{}{\underset{{\emptyset \in w}_{p}}{\sum }}{\left(\emptyset -\emptyset_{p}\right)}^{2}H_{D}\left(\emptyset \right). \end{array}$$

(7) Edge orientation histogram (EOH) aims to build a histogram with the directions of gradients of the edges:

(20) $$\begin{array}{c} \text{EOH}=\tan^{-1}\left(\frac{\text{Vertical}\;\text{Gradient}}{\text{Horizontal}\;\text{Gradient}}\right). \end{array}$$

### 2.3. Classification Using SVM

**Figure 2:**
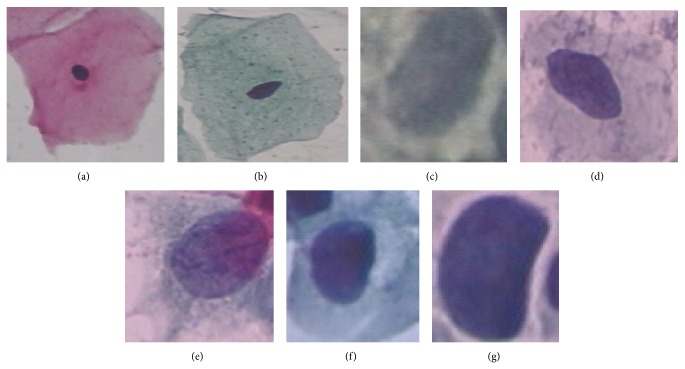
Single cell images “Herlev” data set: (a) normal squamous, (b) intermediate squamous, (c) columnar, (d) mild dysplasia, (e) moderate dysplasia, (f) severe dysplasia, and (g) carcinoma in situ.

The proposed classification system classify the Pap smear images into one of seven classes, namely, superficial squamous, intermediate squamous, columnar, mild dysplasia, moderate dysplasia, severe dysplasia, and carcinoma in situ. The manual classification of the entire dataset has been already done by the experts.  Figure 2 shows sample single cell images in which first row represents three classes of normal image type and the second row represents four classes of malignant type. In the proposed classification system, we used SVM algorithm to classify the Pap smear images. This SVM is based on the work done by Vapnik et al. which is implemented as “LibSVM” [15, 32].

## 3. Experimental Results and Discussion

In the present work, features of Pap smear images such as relative size of nucleus and cytoplasm, dynamic range and first four moments of intensities of nucleus and cytoplasm, relative displacement of nucleus within the cytoplasm, gray level cooccurrence matrix features, local binary pattern histogram, tamura features, and edge orientation histogram are extracted. By the combinations of extracted features, the performance of various classification algorithms are analyzed and compared.

**Figure 3:**
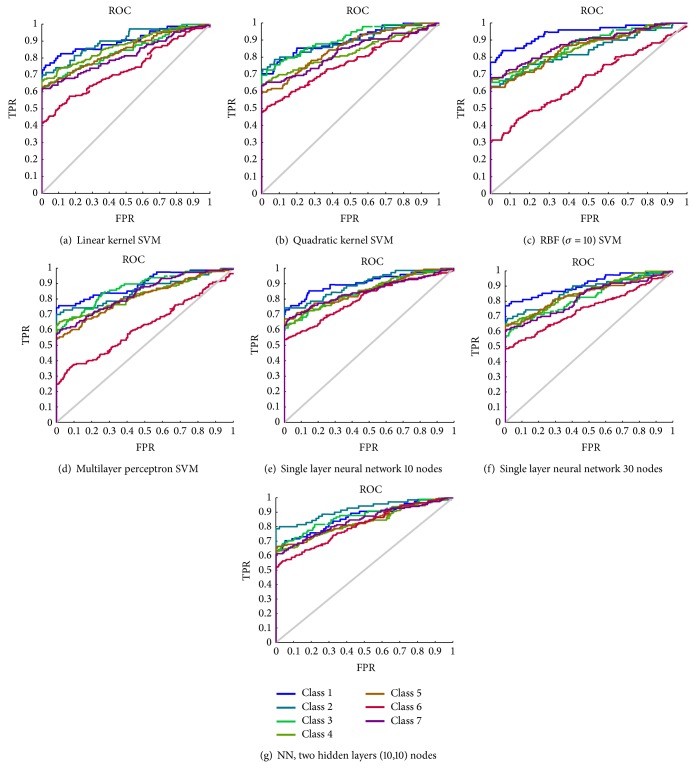
ROC curves for all classes (c1 to c7).

The step by step procedures of the cervical cancer classification method is illustrated as follows.  Table 2 demonstrates the examples of preprocessing steps done in Pap smear using this method. The color images are converted into gray scales and further the segmentation of nucleus and cytoplasm is done in this stage.  Table 3 describes the various features set extracted from the cytology images. Tables 4 and 5 provide the performances measures (precision and recall) for all categories with different combination of features set using SVM classifier. The different classifiers used in this experiment are depicted in Table 7. The classification and diagnostic performance (precision) of the SVM classifiers and its comparison with the NN classifiers are summarized in Table 6. The performance metric and ROC curve for all the classifiers are shown in Figure 3. Area under curve for various classifiers is shown in Table 8. The result of 10-fold cross-validation (confusion matrix) is depicted in Table 9.

In our work, the cell samples are collected from the public database which is free of sampling errors. In order to extract the precise features of the cell nucleus and cytoplasm, the segmentation of cell nucleus from cytoplasm relics a demanding issue. Segmentation is done through morphological filling operations, which is found to be better than the other methods in the detection of nucleus. In this work, the 23 unique features were selected and classified into seven sets, including (1) relative size of nuclei and cytoplasm, (2) dynamic range and first four moments of intensities of nuclei and cytoplasm, (3) relative displacement of Nucleus within the cytoplasm, (4) gray level cooccurrence matrix features, (5) local binary pattern histogram, (6) tamura features, and (7) edge orientation histogram. The result shows that, by using the combinational feature set, it is able to classify all seven types of Pap smear images. It shows that all the dysplasia cells and carcinoma in situ classes have higher nuclear proportion and irregular nucleus and these findings are compatible with the human findings.

The output of SVM shows that the best precision for normal squamous (97.38%), intermediate squamous (93.89%), mild dysplasia (87.33%), severe dysplasia (58.52%), and carcinoma in situ (84.72%) is achieved through the combination of (F1, F2, F3, F4, F5, F6, and F7) feature set. With the single feature set F7, the accuracy rate of 89.35% is achieved in columnar type. Likewise the accuracy of 84.10% for moderate dysplasia is achieved through the combination of F4 and F6 feature set. These observations that show no single feature set produce the best results for all classes. Some feature set shows the predominance results for some classes and as an average, the best overall classification performances were obtained through the combination of all the seven feature sets. Again the recall values of SVM classifiers show that the better performance is obtained through combinational feature set.

The very low precision rate of 58.52% is only observed in severe dysplasia class by any combinations. Most of these image types do not follow the classification rules and it even shows the very poor segmentation results. The need of separate set of classification methods and unique feature selection procedures will improve the performance.

The classification performances of various classifiers are shown in Table 6. The first three classifiers are of SVM based kernel types and the fourth one is the SVM based multilayer perceptron. The other three are neural network based, where the first two use single layer neural network with 10 and 30 nodes and the third uses two hidden layers of (10,10) nodes. The results show that the better performance is achieved through linear kernel SVM classifier than any other classifier. In addition, the performance of the classifiers builds using SVM, outperformance of the NN classifier.

In order to evaluate 917 image instances, 10-fold cross-validation has been done, which hold out a percentage of their data set in each fold to properly evaluate the classifier. The results are shown as confusion matrix (Table 9) with the entire set of features. Out of 917 images, 71/74 normal squamous, 65/70 intermediate squamous, 85/98 columnar, 158/182 mild dysplasia, 121/146 moderate dysplasia, 157/197 severe dysplasia, and 137/150 carcinoma in situ are found to be correct. Except severe dysplasia and carcinoma in citu categories, this method provides compromising outputs.

## 4. Conclusion

In this paper, an improved method for classifying Pap smear images using selected texture image feature is proposed. The study reveals that the method not only helps in classification but also helps in selecting the features which are most suitable for all types of classes. These results show that there is no unique set of feature suitable for all classes. In this classification method, it is concluded from the analysis that the linear kernel SVM classifier out performs than any other classifier.

## Figures and Tables

**Table 1:** The distribution of the 917 cells in Herlev database [22].

Class	Category	Cell type	Cell count	Subtotal
1	Normal	Normal squamous	74	**242**
2	Normal	Intermediate squamous	70	
3	Normal	Columnar	98	

4	Abnormal	Mild dysplasia	182	**675**
5	Abnormal	Moderate dysplasia	146	
6	Abnormal	Severe dysplasia	197	
7	Abnormal	Carcinoma in situ	150	

**Table 2:** Preprocessing steps of Pap smear images.

	Normal squamous	Intermediate squamous	Columnar	Mild dysplasia	Moderate dysplasia	Severe dysplasia	Carcinoma in situ
Original image	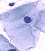	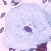	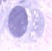	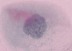	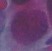	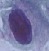	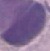

Grayscale image	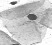	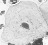	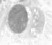	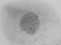	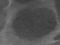	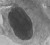	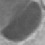

Segmented results	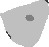	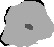	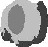	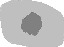	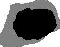	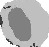	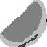

Cytoplasm	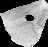	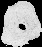	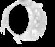	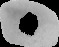	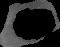	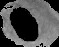	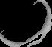

Nucleus	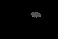	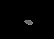	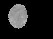	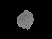	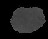	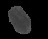	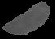

**Table 3:** Feature set description.

Feature set	Features
F1	Relative size of nuclei and cytoplasm
F2	Dynamic range and first four moments of intensities of nuclei and cytoplasm
F3	Relative displacement of nucleus within the cytoplasm
F4	Gray level cooccurrence matrix features
F5	Local binary pattern histogram
F6	Tamura features
F7	Edge orientation histogram

**Table 4:** Precision of SVM classifier for the combination of various features set.

SVM with linear kernel	Precision %						
Features	Normal squamous	Intermediate squamous	Columnar	Mild dysplasia	Moderate dysplasia	Severe dysplasia	Carcinoma in situ
F1	93.67	91.27	89.30	80.13	84.06	32.75	86.46
F2	94.54	89.52	87.34	73.58	84.06	38.43	83.19
F3	91.92	92.36	89.30	80.13	84.06	21.40	83.62
F4	93.67	92.14	87.55	80.13	84.06	27.29	83.62
F5	97.01	92.36	89.03	80.13	84.06	28.17	84.06
F6	96.51	91.92	87.34	80.79	84.06	27.95	83.41
F7	90.61	92.36	89.35	80.13	84.06	23.58	83.62
F1, F2, F3	94.54	89.52	88.43	77.73	84.06	47.38	85.59
F4, F6	96.94	91.92	87.99	80.57	84.10	29.91	83.62
F4, F5, F6	96.07	91.27	86.90	79.69	83.62	36.90	84.50
F4, F5, F6, F7	96.29	91.27	85.59	78.82	83.84	45.41	84.28
F1, F2, F3, F4, F5, F6, F7	97.38	93.89	86.90	87.33	83.62	58.52	84.72

**Table 5:** Recall of SVM classifier for the combination of various features set.

SVM with linear kernel	Recall %						
Features	Normal squamous	Intermediate squamous	Columnar	Mild dysplasia	Moderate dysplasia	Severe dysplasia	Carcinoma in situ
F1	90.17	86.71	82.39	76.18	83.56	28.52	82.86
F2	90.46	85.50	85.91	71.19	82.88	31.67	79.09
F3	90.91	88.16	85.13	78.11	82.03	20.42	81.69
F4	89.71	89.23	82.56	78.94	82.16	23.23	82.99
F5	93.81	89.31	83.83	79.78	83.89	25.79	82.40
F6	87.86	88.76	82.67	80.11	80.96	24.90	82.89
F7	87.67	87.37	85.23	79.09	80.17	20.78	80.12
F1, F2, F3	90.40	85.88	84.13	75.65	81.87	45.35	82.19
F4, F6	90.46	86.67	84.49	75.34	81.03	27.80	82.09
F4, F5, F6	89.89	85.17	87.10	78.61	81.72	34.51	83.02
F4, F5, F6, F7	92.29	87.87	82.59	75.00	80.78	43.50	82.78
F1, F2, F3, F4, F5, F6, F7	91.59	89.71	80.35	85.78	80.02	53.48	83.79

**Table 6:** Precision of various classifiers for different classification of cervical cytology images.

Classifiers	Precision %						
	Normal squamous	Intermediate squamous	Columnar	Mild dysplasia	Moderate dysplasia	Severe dysplasia	Carcinoma in situ
C1	96.91	93.89	92.35	92.33	96.62	92.10	91.72
C2	94.76	91.92	87.99	79.26	78.60	63.10	83.41
C3	97.60	93.23	86.68	86.24	83.84	44.32	86.24
C4	91.92	90.17	82.31	79.48	79.91	34.93	75.76
C5	91.92	96.07	85.59	84.06	79.48	74.45	78.82
C6	95.41	91.92	81.00	84.50	81.66	68.12	79.04
C7	95.85	91.92	89.97	85.59	94.10	89.98	90.18

**Table 7:** Various classifiers used.

Classifier	Description
C1	Linear kernel SVM
C2	Quadratic kernel SVM
C3	RBF (σ = 10) SVM
C4	Multilayer perceptron SVM
C5	Single layer neural network 10 nodes
C6	Single layer neural network 30 nodes
C7	NN, two hidden layers (10, 10) nodes

**Table 8:** Area under curve for various classifiers.

Classifier	Normal squamous	Intermediate squamous	Columnar	Mild dysplastic	Moderate dysplastic	Severe dysplastic	Carcinoma in situ
C1	0.9488	0.9187	0.8466	0.8631	0.8459	0.7544	0.8450
C2	0.8986	0.9283	0.8513	0.8313	0.8519	0.7518	0.8374
C3	0.9202	0.8666	0.8474	0.8337	0.8568	0.6807	0.8427
C4	0.8948	0.8855	0.8249	0.8249	0.8268	0.6556	0.7881
C5	0.8881	0.8856	0.8705	0.8371	0.8261	0.8290	0.8286
C6	0.8864	0.8551	0.8270	0.8531	0.8341	0.7970	0.8210
C7	0.8431	0.8956	0.8322	0.8986	0.8399	0.8070	0.8424

**Table 9:** Confusion matrix of 10-fold cross-validation using linear kernel SVM with the entire set of features.

	Normal squamous	Intermediate squamous	Columnar	Mild dysplasia	Moderate dysplastic	Severe dysplastic	Carcinoma in situ
Normal squamous	**71**	2	1	0	0	0	0
Intermediate Squamous	3	**65**	1	1	0	0	0
Columnar	1	7	**85**	5	0	0	0
Mild dysplasia	1	7	11	**158**	4	1	0
Moderate dysplasia	0	0	1	7	**121**	13	4
Severe dysplasia	0	1	3	12	32	**157**	35
Carcinoma in situ	0	0	0	3	7	13	**137**
